# A223 DEMOGRAPHIC AND CLINICAL CHARACTERISTICS OF PATIENTS WITH INFLAMMATORY BOWEL DISEASE AND PRIMARY SCLEROSING CHOLANGITIS AT THE OTTAWA HOSPITAL

**DOI:** 10.1093/jcag/gwad061.223

**Published:** 2024-02-14

**Authors:** Y Alrifae, G Sambhi, E I Benchimol, A Ricciuto, S Murthy

**Affiliations:** University of Ottawa, Ottawa, ON, Canada; University of Ottawa, Ottawa, ON, Canada; University of Toronto, Toronto, ON, Canada; University of Toronto, Toronto, ON, Canada; University of Ottawa, Ottawa, ON, Canada

## Abstract

**Background:**

Primary sclerosing cholangitis (PSC) is present in up to 8% of individuals with inflammatory bowel disease (IBD). Characteristics of this population are unique and data are limited.

**Aims:**

To describe the demographic and clinical characteristics of all individuals with PSC-IBD managed at The Ottawa Hospital (TOH).

**Methods:**

A chart review was performed for 132 patients with PSC-IBD followed at TOH between January 1998 and July 2023.

**Results:**

Of 132 individuals with PSC-IBD identified at TOH, 26.5% had Crohn’s disease (CD), 68.2% had ulcerative colitis (UC) and 5.3% had IBD-unclassified. Median age at diagnosis were 25.3 (IQR 20.94) for IBD and 33.84 (IQR 23.98) for PSC, and 66.7% of all patients were male. More than 17% had a co-morbid autoimmune disease other than IBD or PSC. Of those with CD, roughly 15% had fibrostenotic or penetrating complications and 17% had perianal disease at the time of IBD diagnosis. Only 28% ever had a liver biopsy, whereas the majority were diagnosed with PSC by cholangiography. PSC distribution was isolated intrahepatic in 47%, isolated extra-hepatic in 5.1% and mixed intra/extra-hepatic in 38.5%. Clinically significant portal hypertension was present in 11.5% at PSC diagnosis, while 27.3% and 16.7% developed cirrhosis and liver decompensation, respectively by last follow-up. 17.4% underwent liver transplant, of which 30.4% developed PSC recurrence post-transplant.

**Conclusions:**

Our study showed that the majority of PSC-IBD patients are young males with UC. Co-morbid autoimmune diseases are common while isolated extra-hepatic PSC is uncommon. Cirrhosis, liver decompensation and liver transplant are frequent occurrences in this population. PSC recurrence post-transplant is observed in more than 30% of patients.

**Funding Agencies:**

None

